# A Multilevel Probabilistic Beam Search Algorithm for the Shortest Common Supersequence Problem

**DOI:** 10.1371/journal.pone.0052427

**Published:** 2012-12-27

**Authors:** José E. Gallardo

**Affiliations:** Departamento de Lenguajes y Ciencias de la Computación, Universidad de Málaga, Málaga, Spain; Universitat Rovira i Virgili, Spain

## Abstract

The shortest common supersequence problem is a classical problem with many applications in different fields such as planning, Artificial Intelligence and especially in Bioinformatics. Due to its NP-hardness, we can not expect to efficiently solve this problem using conventional exact techniques. This paper presents a heuristic to tackle this problem based on the use at different levels of a probabilistic variant of a classical heuristic known as Beam Search. The proposed algorithm is empirically analysed and compared to current approaches in the literature. Experiments show that it provides better quality solutions in a reasonable time for medium and large instances of the problem. For very large instances, our heuristic also provides better solutions, but required execution times may increase considerably.

## Introduction

In this section, the shortest common supersequence problem is introduced. We formally state the problem, describe its complexity and summarize previous approaches that have been reported in the literature to tackle this problem.

### The Shortest Common Supersequence Problem

The shortest common supersequence problem (SCSP) is a classical problem in stringology. In order to state this problem, let 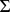 denote a finite set of symbols (the alphabet), and let a string 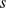 taken from such an alphabet (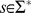) be a finite sequence of zero or more of those symbols. We write 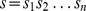 (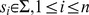) to denote such a string as a sequence of 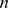 symbols, 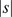 for the length of string 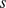 and 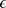 for the empty string. We write 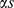 (where 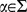) for the string obtained by prepending the symbol 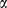 in front of string 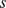, and 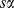 for appending symbol 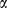 at the end of 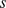. Abusing the notation, we will also write 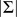 for the cardinality of the alphabet and 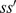 (where 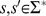) for the concatenation of both strings.

Let us now consider two strings 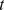 and 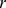 taken from 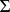. String 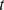 is said to be a supersequence of 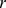 (denoted as 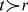) if the following recursive definition holds:
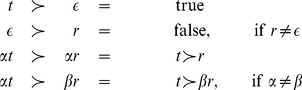
(1)


In other words, 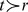 implies that 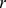 can be embedded in 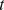, meaning that all symbols in 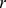 are present in 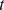 in the very same order (although not necessarily consecutive). For example, given the alphabet 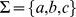, 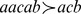.

We can now state the SCSP as follows: an instance 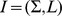 for the SCSP is given by a finite alphabet 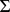 and a set 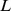 of 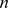 strings 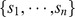 (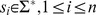). Solving this instance consists of finding a string 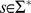 of minimal length that is a supersequence of each string in 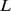 (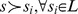 and 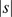 is minimal). For example, given 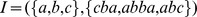, a shortest common supersequence of 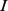 is 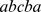.

During the construction of a solution to the SCSP the following function will be useful to assess the progress of the algorithm:
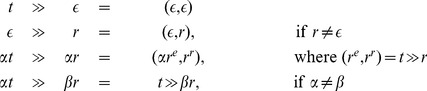
(2)


Intuitively, 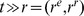 if 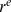 is the longest initial segment of 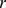 embedded by 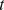, and 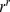 is the remaining part of 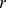 not embedded by 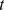 (for example, 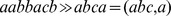). Note that 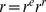, and 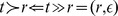. Let 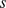 be a (not necessarily complete) candidate solution to SCSP instance 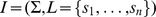, and let 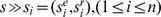. We write 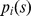 (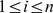) to denote 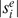 (i.e. the length of the longest prefix of 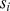 embedded by 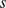) and 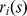 to denote 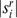 (the length of the remaining part of 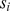 not embedded by 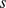). We also write 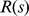 to denote set 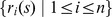.

From a practical point of view, the SCSP is a very interesting combinatorial problem as it has many applications in different fields such as planning [1] or data compression [2] in Artificial Intelligence and especially in Bioinformatics, where it is closely related to the problem of multiple sequence alignment [3] and is also used in the production of oligonucleotide microarrays, a fundamental tool in Genomics [4]–[6]. These oligonucleotide arrays are manufactured using a variant of the photolithographic method used in the semiconductor industry, where nucleotides are added to the microarray in a series of so called cycles. The fabrication cost and time for a oligonucleotide array largely depend on the number of cycles, as each cycle is a laborious process that takes about 5 minutes to complete. In addition, current production processes are not perfect, and the probability for fabrication errors is increased with the number of cycles. Therefore, it is very important to produce oligonucleotide arrays in as few cycles as possible. This optimization problem is known as the synthesis strategy problem, and was shown to be equivalent to the SCSP [7]. As a consequence, in this context, even a small reduction in the length of attained supersequences leads to significant benefits.

The SCSP problem has been shown to NP-hard in general [8], and remains so even after imposing some constraints on 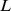 or 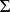. For example, it is NP-hard in general when all 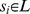 have length two [9], or when the alphabet size is two [10]. In addition, the problem is not fixed-parameter tractable for many practical parametrizations [11], [12], and this also implies the absence of FPTAS (fully polynomial-time approximation schemes) for the corresponding problem. As a consequence, we can not expect to efficiently solve the SCSP using conventional exact techniques (unless 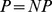) and have to resort to tackling the problem by means of heuristics.

### Previous Approaches

Regarding classical exact techniques, the SCSP can be approached by means of Dynamic Programming [13], but memory requirements of the resulting algorithm grows exponentially with the number of strings in the undertaken instance [14]. The problem can also be solved in an exact way by using a Branch and Bound algorithm [15], but such an approach is neither practical as its complexity is exponential in the size of the alphabet [14]. Nevertheless, heuristic variants of Branch and Bound have been used to effectively tackle large instances of the SCSP. We will review more thoroughly these algorithms below.

A popular greedy constructive heuristic for the SCSP is Majority Merge [16] (MM), that incrementally constructs a supersequence by adding the symbol most frequently found at the front of the strings in 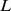, and removing these symbols from the corresponding strings. A variant of this algorithm that concentrates in shortening longer strings firstly is known as Weighted Majority Merge (WMM). This algorithm is similar to MM, but a weight (that depends on the length of the string in whose front it is located) is assigned to each symbol, and the symbol with higher weight is added to the constructed supersequence. Alphabet Leftmost [4] is another algorithm that takes as input a permutation of all symbols in the alphabet. The algorithm then proceeds with successive repetitions of this permutation until all the strings in the instance are embedded, although unproductive steps (i.e. those for which the next symbol in the row does not appear at the front of any string) are discarded.

Different metaheuristics have also been used to tackle the SCSP, like an Evolutionary Algorithm (EA) [17] that evolves weights to be used by WMM [16], [18], various EAs and Memetic Algorithms (MA) [19] endowed with local search proposed by Cotta [20], [21], an Ant Colony Optimization (ACO) [22] algorithm introduced by Middendorf [23], and a so called POEMS (Prototype Optimization with Evolved Improvement Steps) algorithm by Kubalk [24].

Ning et al. compare different greedy algorithms with lookahead on random and real DNA instances of the SCSP with thousands of nucleotides [5]. A post-processing algorithm was also used on resulting strings in order to reduce their length further. Subsequently, a Deposition and Reduction (DR) algorithm was devised for very large SCSP instances consisting of many long DNA and protein sequences [6]. In this work, the deposition process is responsible for generating a small set of common supersequences, and the reduction process (an extension of the one presented by Ning et al. [5]) shortens these supersequences.

A heuristic variant of Branch and Bound algorithm, known as Beam Search (BS) [25], [26], was firstly applied to the SCSP by Gallardo et al. [27]. Beam Search performs an incomplete breadth first search on the tree corresponding to the solution space (i.e. each node in a level of the tree is explored before moving on to the next level), keeping only the most promising (according to some quality estimation) partial solutions on each level of the exploration, and discarding those remaining without guaranteeing that they do not lead to an optimal solution. Solutions kept on each level of the exploration constitute the so called *beam*, and the maximum number of such solutions is a parameter of the algorithm denoted as the beam size. In addition, a lower bound is computed for each partial solution, and nodes that can not improve the incumbent solution are also pruned. In the same work, a collaborative hybridization of BS algorithm with a MA was shown to outperform constituent algorithms. Later, a Probabilistic Beam Search (PBS ) algorithm for the SCSP was presented [28]. Instead of strictly selecting more promising solutions on each level of the exploration, this algorithm uses a probabilistic approach for including elements in the beam.

Recently, an enhanced Beam Search algorithm (IBS) that performs at the state of the art for this problem has been presented [29]. In that paper, experimental evidence was provided to show that IBS is able to better find quality solutions for random and real instances of the SCSP than all previous approaches in the literature. The differentiating characteristics of IBS algorithm are:

- It uses Dynamic Programming to pre-compute a table with information that can be used during the execution of BS procedure in order to accurately estimate the quality of different partial solutions. More precisely, as a heuristic to evaluate partial solution 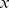, the probability (assuming that strings in 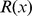 are independent) of 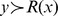 (where 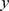 is a random string) is used. For this purpose, the following recurrence

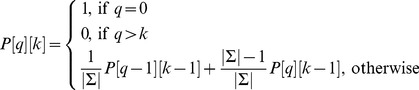
(3)can be used to populate a two-dimensional array 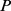 such that 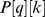 stores the probability 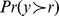, where 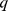 is the length of string 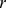 and 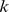 is the length of random string 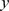 (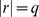 and 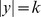), and the heuristic value (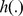) for partial solution 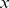 can be computed as:
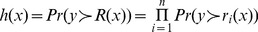
(4)

- A technique called *dominance pruning* is used to further reduce the explored search tree during BS. For this purpose, the 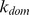 best candidate solutions in the beam are chosen as dominators, and any other solution that is dominated by any of them is discarded (a candidate solution 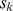 is dominated by another candidate solution 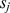 if 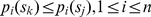).
- With the aim of reducing execution times, the algorithm does not compute lower bounds for partial solutions.

### This Work

In this work we present a new multilevel PBS heuristic (denoted as MPBS ) for the SCSP that uses PBS algorithm at different levels. MPBS is experimentally compared to other algorithms, and especially to IBS [29], that constitutes currently the state of the art for the SCSP. Results show that MPBS can obtain better results than IBS on all tested real and random instances. MPBS is inspired by IBS, but it incorporates the following innovations:

- Contrary to IBS, MPBS is a probabilistic algorithm and thus can provide different solutions to the same instance of a problem on different runs.
- Various Probabilistic Beam Search procedures are used in the resulting algorithm at different levels. On the one hand, one instance of PBS is used as a constructive algorithm to obtain an initial solution. This is a typical way of using such an algorithm. On the other hand, two other PBS procedures are used to further reduce solutions constructed like this. These reduction algorithms show how PBS can be used as a local searcher. We do not currently know of any other work where this approach has been considered.

## Materials and Methods

In this section, the multilevel Probabilistic Beam Search Algorithm (MPBS ) is described. Firstly, a generic description of the PBS algorithm is presented. Afterwards, different components of the algorithm based on PBS are described along with the resulting algorithm that integrates all of them.

### Probabilistic Beam Search

In this section we describe the Probabilistic Beam Search Algorithm (PBS) for the SCSP. This algorithm is later used at different levels as a component of the proposed MPBS heuristic.

**Figure 1:**
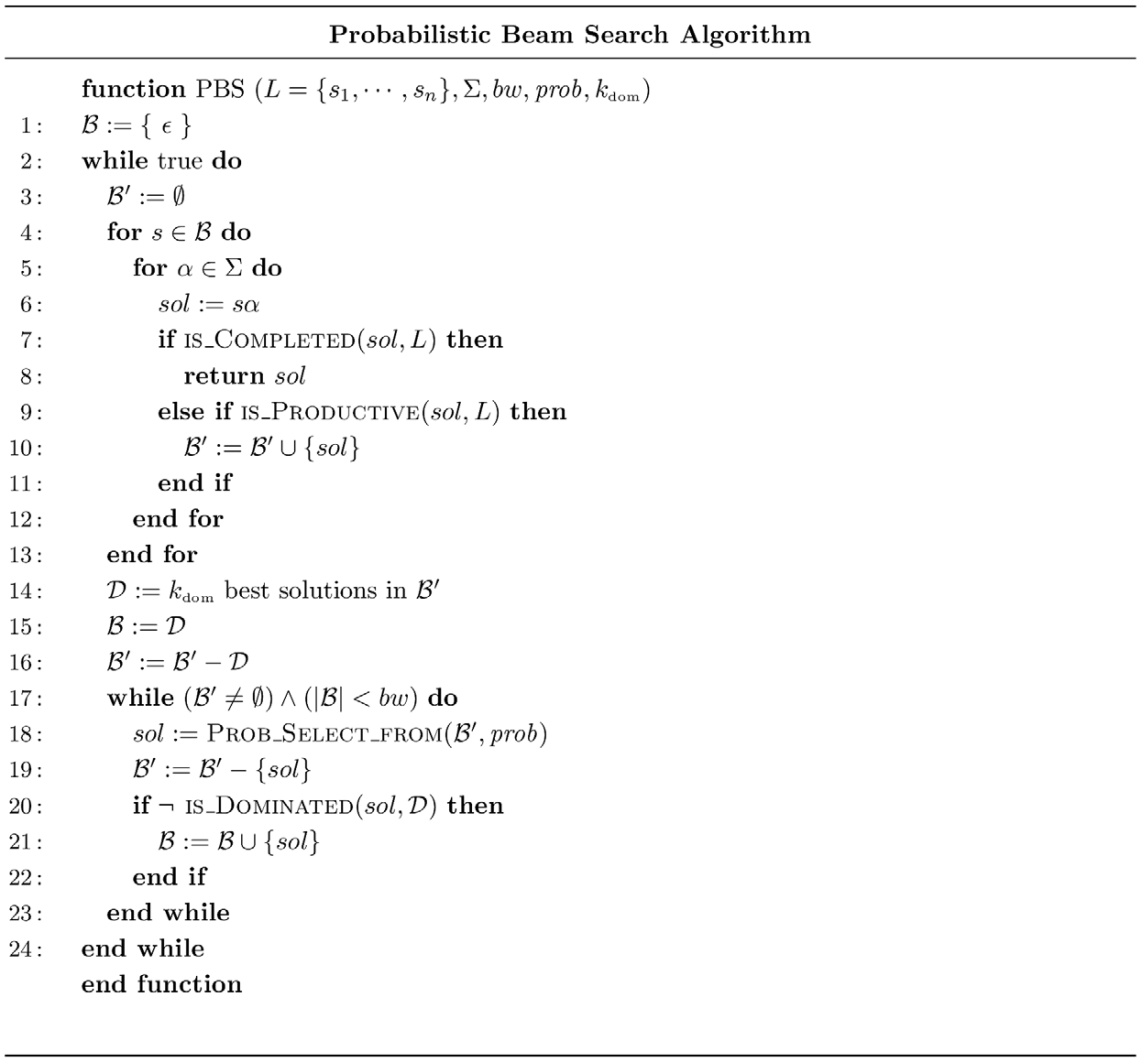
Probabilistic Beam Search Algorithm.

PBS is a variant of Beam Search algorithm and its pseudo code is shown in Figure 1. Like BS, it performs an incomplete breadth first exploration of the search space, but instead of strictly keeping best nodes on each level of the exploration tree, nodes are selected in a probabilistic way. More precisely, PBS works as follows: At each step of the algorithm, a set 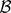 of partial solutions – called the *beam* – is maintained. At the start of the algorithm, 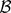 only contains the empty partial solution (that is, 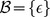). On each iteration of the algorithm, all partial solutions in the beam are extended with each of the symbols in the alphabet. If any of those tentative solutions constitutes a solution to the instance (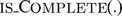 predicate), said solution is returned, and the algorithm terminates. Otherwise, if a new symbol just appended to the solution contributes to embedding a new symbol for any string in 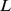 (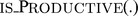 predicate), the extended solution is included in the auxiliary beam (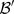). Afterwards, up to 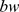 (a parameter of the algorithm standing for the *beam width*) solutions from 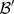 are included in the beam for the next iteration. For this purpose, partial solutions are ranked according to their heuristic value 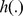 (Equation 4 ) and a set of dominators (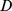) with the best 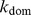 solutions are selected and included in the new beam. The rest of solutions are probabilistically selected (using 
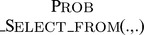
 function) as follows: First, a random real number 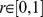 is drawn. If 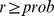 (where 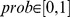 is a parameter of the algorithm controlling randomness of selection), the solution in 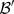 with higher heuristic value is chosen (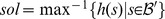). Otherwise, linear ranking [30] is used to choose the solution. In any case, *dominance pruning* is performed, and the selected solution is discarded in case it is dominated by any of the 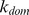 dominators.

### Reduction via PBS

**Figure 2:**
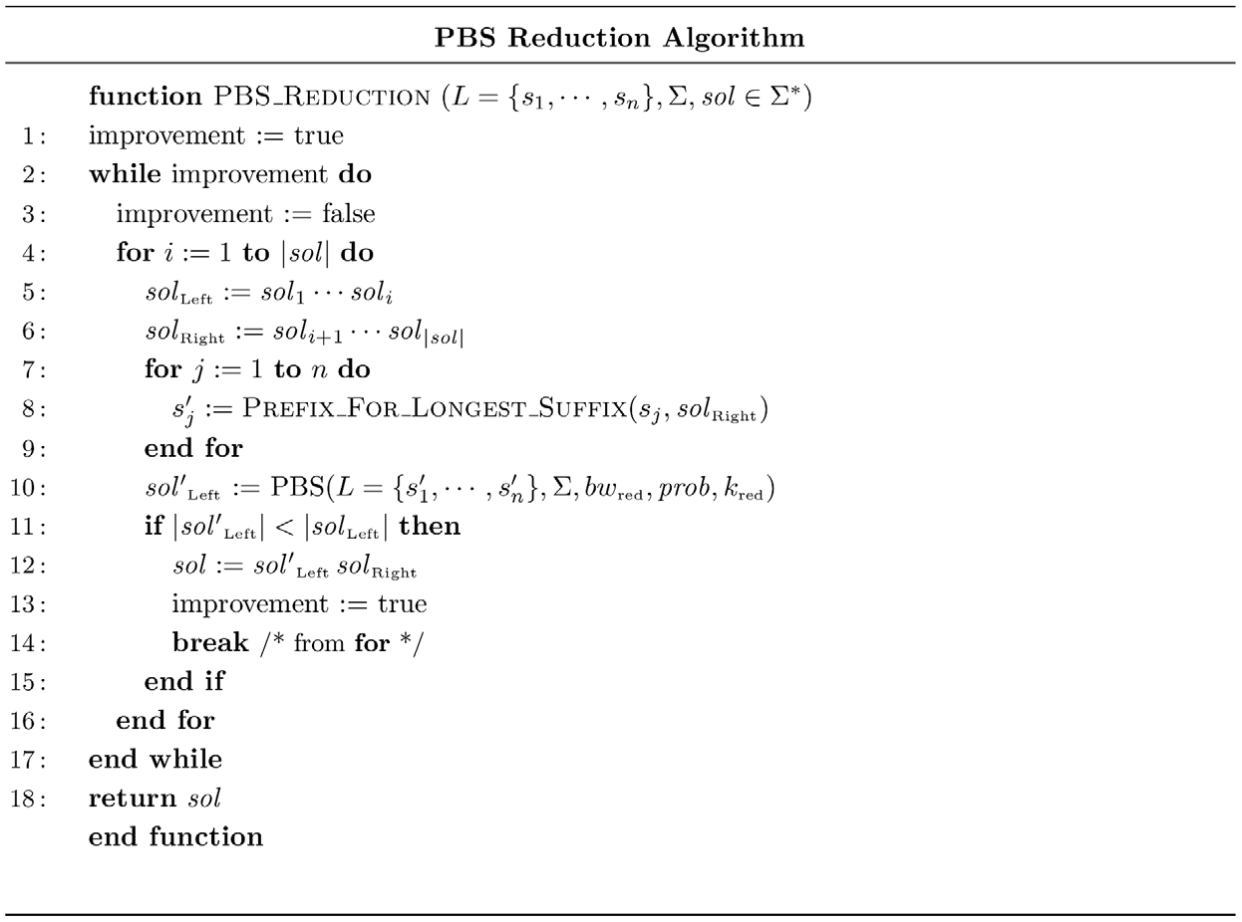
Reduction Algorithm.

In the previous section we described how PBS can be used as a constructive heuristic for the SCSP. In this section we describe how it can be embedded into a reduction algorithm in order to implement a local search procedure. This reduction algorithm is inspired by the one proposed by Ning et al. [5] and the corresponding pseudo code is shown in Figure 2.

In order to describe the functioning of this algorithm, let us firstly depict function 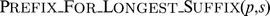. For this purpose, let 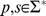, and let 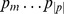 denote the longest suffix of 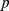 that is a subsequence of 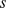. Then, 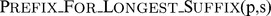 returns 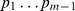, i.e. the prefix of 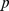 that is not part of the longest suffix of 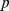 that is subsequence of 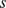. In the pseudo code, 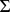 and 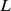 define the undertaken SCSP instance and 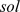 stands for a common supersequence to the instance that we aim to shorten as follows: current solution 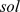 is split into a prefix (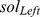) and a suffix (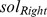), longest suffixes that can be embedded in 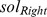 are computed for all strings in 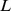 and a new instance of the problem is constructed with corresponding non-embedded prefixes. This instance is solved using PBS yielding a supersequence (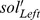) for non-embedded prefixes, and, if an improvement has been achieved with respect to the prefix of the original solution (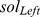), that prefix is substituted with 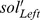. This process is attempted for all possible splits of current solution, and if an improvement is made it is restarted with enhanced solution.

### Perturbation via PBS

A perturbation local search algorithm for the SCSP that uses PBS as a repairing procedure is presented in this section.

**Figure 3:**
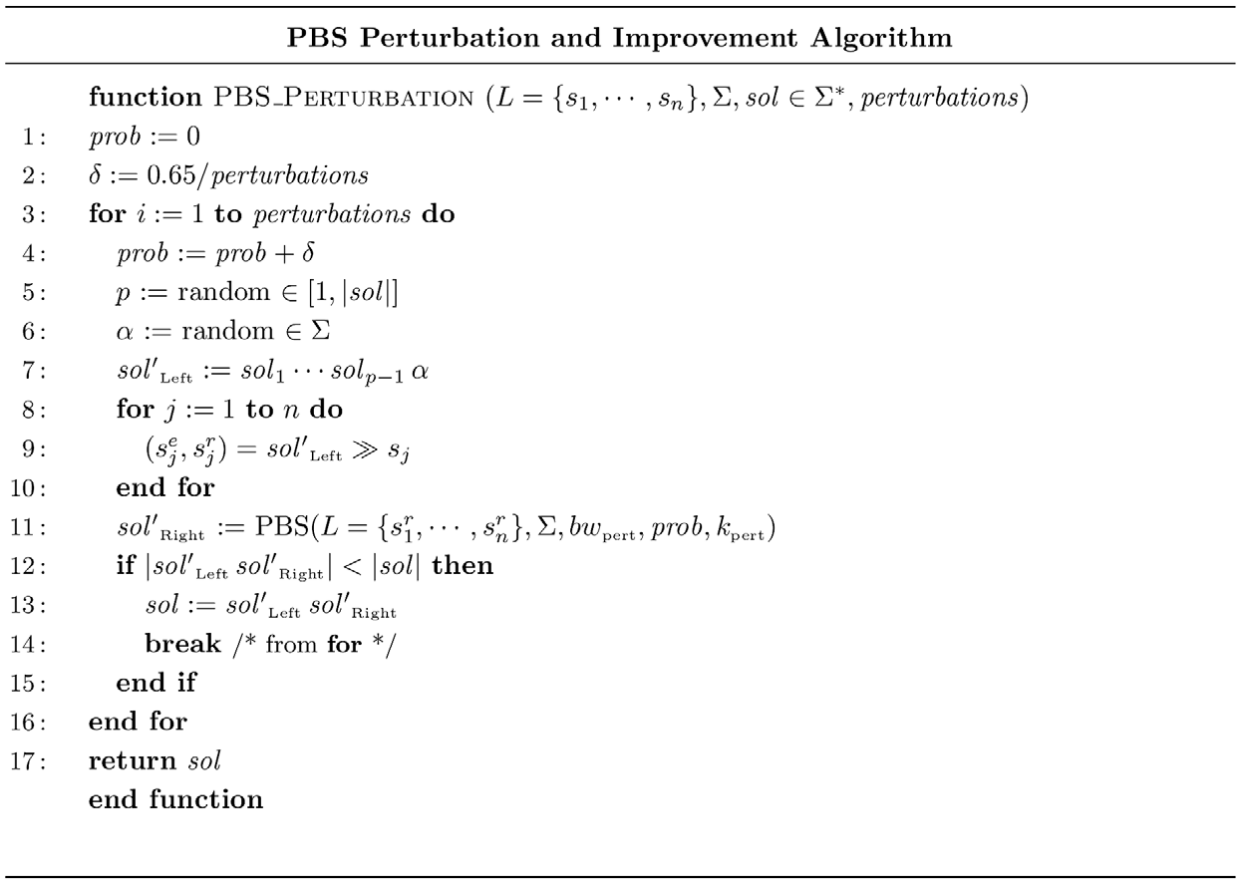
Perturbation and Improvement Algorithm.

In order to perturb a solution, an arbitrary symbol in it can be replaced by a different one. This perturbation will usually lead to an infeasible solution (i.e. a sequence that is not a supersequence any more) and, therefore, a repairing mechanism must subsequently be applied. The precise pseudo code for this algorithm is shown in Figure 3, where 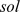 stands for an initial solution to 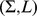 instance, 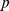 is the position of the symbol to be mutated and 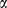 is new symbol to replace 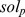. As shown, 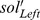 corresponds to the prefix of mutated sequence that includes all symbols up to 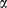. This prefix will not in general embed all strings in 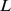, hence a new instance with suffixes from 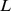 that are not embedded by 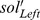 is defined and PBS algorithm is used to solve it. The obtained supersequence (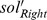) is concatenated to 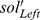, yielding a new feasible supersequence for the original instance. Note that the new supersequence can be shorter than the original one and in this case it will be returned. This perturbation and improvement process is attempted for a number of iterations (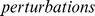), and the randomness (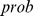) of selection in the PBS algorithm used for repairing is linearly increased with each iteration of the algorithm (from 0% in first iteration to 65% in the last one). By increasing this parameter, partial solutions with lower heuristic value get more chances to be included in the beam and, as a consequence, search is directed towards alternative regions of the search space. Hence, the algorithm starts by exploring solutions that are close to the current one, but if no improvement is achieved, dissimilar solutions are considered.

### Multilevel PBS Algorithm

**Figure 4:**
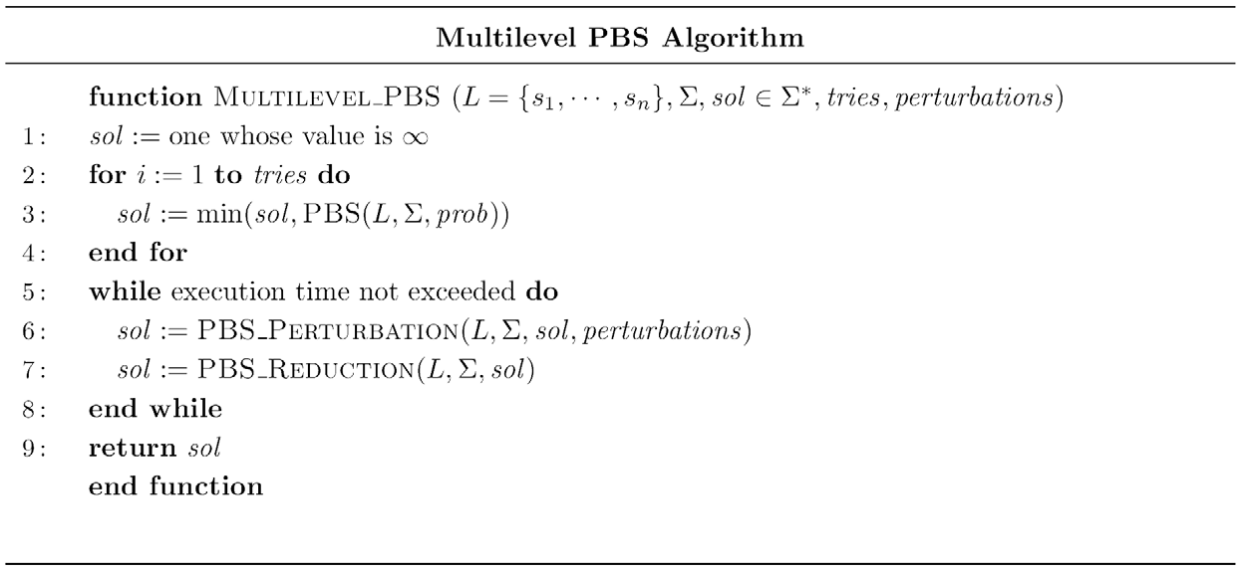
Multilevel Probabilistic Beam Search Algorithm.

We are now in a position to describe the Multilevel PBS algorithm, whose pseudo code is shown in Figure 4. The algorithms starts by performing several executions of PBS algorithm with the aim of obtaining a good quality initial solution. Afterwards, until the allowed execution time is not reached, PBS_Reduction and PBS_Perturbation processes are alternatively performed on the current solution, and any improvement so achieved in the quality of the solution is recorded. Finally, the best solution found is returned.

## Results and Discussion

In order to evaluate the different heuristics, two sets of benchmarks have been considered (all instances are available at http://www.lcc.uma.es/pepeg/scs_instances.zip).

The first one – henceforth referred to as RandomSet – is composed of random strings with different lengths and has been used as a benchmark for different algorithms in the literature [21], [27]–[29]. For this benchmark, each instance is composed of eight strings, four of them with 40 symbols, and the remaining four with 80 symbols. Each of these strings is randomly built, using an alphabet 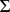. Five subsets of instances have been defined using different alphabet sizes, namely 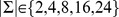. For each alphabet size, five different instances have been generated. Thus, the benchmark set consists of 25 different problem instances.

The second set of instances – referred to as LargeRealSet – consists of instances with a large number of lengthy strings taken from DNA and protein sequences, and was originally used by Ning and Leong [24]. We have considered 6 DNA datasets and 3 protein datasets, each one comprising 10 different instances. Instances of each dataset are characterized by a number of strings (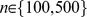) of the same length (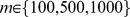).

Our algorithm (MPBS ) was coded in C and compiled using gcc 4.1.2. Experiments were performed on a HP Proliant SL170s computer (2 Intel Xeon X5660 2.8 GHz, 8GB RAM) running CentOS 5.5 Linux. As for different parameters, they were set as follows: 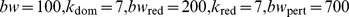 and 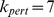 (see next subsection for a sensibility analysis of these parameters).

For the purpose of comparing MPBS to other algorithms, we have also run them on the same machine. The implementations of MA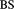 [27] and PBS [28] were available to us. The implementation of IBS [29] was provided by their authors but was written in Java. In order to better compare running times, we coded IBS in C. Finally, implementation of DR [6] was not available, so we coded it also in C. During the deposition and reduction phases of this algorithm, Majority Merge heuristic with a lookahead of 3 steps was used, as described by DR authors. In this way, all algorithms compared in this work were compiled using same compiler and settings as MPBS.

In order to report results in tables and figures, we always calculate the relative percentage difference (RPD) of solutions obtained by different algorithms from best-known solution for corresponding instance (defined as 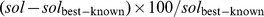), and provide statistical values for those differences. Best-known solutions for each instance are available with the instances, so reproducibility of the results is possible in the future.

For instances in RandomSet, a maximum execution time of 300 seconds per instance was allowed, and 20 different runs for each instance were executed (thus, 100 runs for each alphabet size), except for DR and IBS that are deterministic algorithms and hence provide a single solution to each instance. Results of these experiments are shown in Table 1 as the mean and standard deviation for RPD from best-known solutions of results obtained by each algorithm. It can be observed that PBS performs better than MA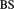, except for 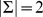, and that DR obtains worse results. Best results for these instances are achieved by MPBS that outperforms all other approaches, followed by IBS. A non-parametric *Mann-Whitney-Wilcoxon* test [31], [32] indicates that results of MPBS are statistically significant (at the standard 5% level) with respect to the ones produced by IBS for all instances and alphabet sizes, except for 2 (out of 25) instances (one with 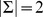 and another one with 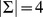). In order to better compare these algorithms, second column of Table 2 reports mean relative improvement of solutions obtained by MPBS with respect to the ones obtained by IBS, showing that the improvement in solution quality is more noticeable for instances with a larger alphabet size. Next column in this table shows that MPBS was able to obtain a solution better than or equal to the one provided by IBS in all instances and executions for this dataset. Finally, the time (in seconds) needed by IBS to produce solutions for these instances is compared to the time needed by MPBS to reach such a solution. It can be observed that MPBS needs more time to achieve solutions with same quality as those provided by IBS, but the average time needed is less than 7.10 seconds in all cases.

**Table 1:** Experimental results for RandomSet instances.

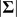	MA 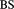 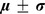	PBS 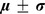	DR 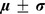	IBS 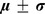	MPBS 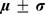
2	1.59±0.97	2.371.23	6.42±1.48	0.55±0.44	0.00±0.00
4	4.68±1.22	4.29±0.68	16.24±1.82	2.30±1.39	0.45±0.56
8	10.04±2.44	6.11±1.36	19.80±4.04	2.61±1.36	0.67±0.57
16	8.82±1.42	7.64±1.01	25.20±0.29	4.91±1.16	1.36±0.64
24	11.78±2.25	9.59±1.56	28.36±2.11	6.50±1.69	1.93±0.83

Results obtained by MA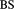, PBS, DR, IBS and MPBS for instances in RandomSet. Different alphabet sizes have been considered (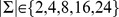) and, for each alphabet size, a dataset with 5 different instances has been defined. Table shows statistical values (mean (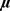) and standard deviation (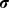)) for relative percentage difference (RPD) from best-known solutions of solutions obtained by each algorithm.

**Table 2:** MPBS and IBS comparison for RandomSet instances.

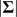	MPBS Impr.	P( 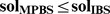 )	IBS Time 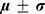	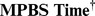 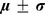
2	0.55	100%	0.09±0.01	0.18±0.04
4	1.85	100%	0.23±0.01	0.43±0.17
8	1.94	100%	0.60±0.02	1.80±1.67
16	3.55	100%	1.30±0.07	7.09±6.91
24	4.57	100%	1.81±0.08	5.64±6.09

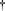
Time(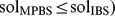.

Second column shows mean improvement of solutions obtained by MPBS with respect to the ones obtained by IBS for instances in RandomSet with different alphabet sizes (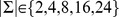). Third column shows the percentage of MPBS runs that were able to reach a solution better than or equal to the one provided by IBS. Fourth column shows statistical values (mean (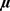) and standard deviation (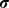)) for the time (in seconds) needed by IBS to produce its solution. Fifth column shows statistical values (mean and standard deviation) for the time (in seconds) needed by MPBS to produce a solution better than or equal to the one provided by IBS.

**Figure 5:**
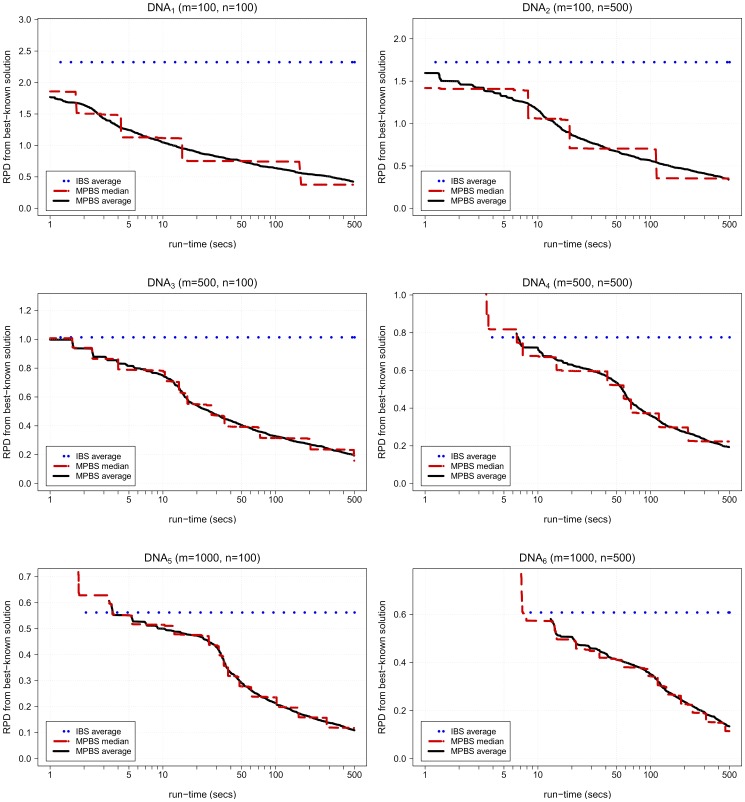
Solution quality over time for different DNA datasets in LargeRealSet. Figure shows evolution along execution time of statistical values (average and median) for relative percentage difference (RPD) from best-known solutions of solutions obtained by MPBS algorithm. Average solution obtained by IBS is also shown. Note that the value of solution is considered to be 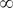 before an algorithm has provided its first solution, hence beginnings of average curves indicate the moment at which at least one solution has been obtained for all executions and instances in the dataset.

For DNA instances in LargeRealSet, a maximum execution time of 500 seconds per instance was allowed, and 20 different runs for each instance were executed for non-deterministic algorithms (recall that each subset consists of 10 different instances, hence 200 executions per subset have been carried out). Results of these experiments are shown in Table 3, where n/a indicates that corresponding algorithm was unable to produce a solution to corresponding instance within allowed execution time. Note that MA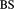 is not able to produce solutions except for the first two subsets. This is due to several reasons like the fact that MA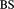 needs to compute a lower bound for each partial solution in the beam and the execution costs of the Memetic Algorithm that cannot effectively make progress for these very large instances. PBS is able to obtain results for all instances except for the ones in the last subset (this algorithm also needs to compute lower bounds for partial solutions in the beam and uses a costly look-ahead strategy in order to assign them an heuristic value). DR is able to solve all instances (recall that this is an algorithm specifically devised for very large SCSP instances), but its solutions are worse than the ones provided by PBS in all but one set. Best results are again obtained by MPBS followed by IBS. A non-parametric *Mann-Whitney-Wilcoxon* test indicates that results of MPBS are statistically significant (at the standard 5% level) with respect to the ones produced by IBS for all instances except for 1 instance in second subset (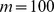 and 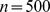). Table 4 compares MPBS to IBS on these instances. It can be observed that MPBS is able again to achieve solutions with at least same quality as those produced by IBS in all executions, and that the average execution time needed for this is less than 23 seconds. Best improvements by MPBS are obtained for instances with shorter strings (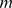 = 100) and those improvements are less than 2% in all cases. In order to show the anytime behaviour of MPBS, Figure 5 represents the evolution along time of solution quality provided by this algorithm (as RPD from best-known solutions) for different datasets in this group of instances. The average solution provided by IBS is also plotted for reference purposes.

**Table 3:** Experimental results for DNA LargeRealSet instances.

Inst.	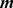	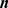	MA 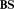 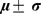	PBS 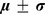	DR 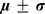	IBS 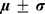	MPBS 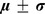
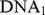	100	100	5.39±1.22	4.15±0.75	5.88±0.92	2.32±0.50	0.42±0.32
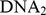	100	500	3.79±0.93	3.75±1.06	3.14±0.65	1.72±0.59	0.34±0.26
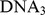	500	100	n/a	4.32±0.29	4.73±0.71	1.01±0.24	0.20±0.11
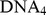	500	500	n/a	3.62±0.57	4.59±1.17	0.77±0.21	0.19±0.12
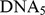	1000	100	n/a	4.43±0.40	5.83±1.02	0.56±0.13	0.11±0.02
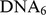	1000	500	n/a	n/a	4.33±0.68	0.61±0.19	0.13±0.02

Results obtained by MA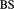, PBS, DR, IBS and MPBS for DNA instances in LargeRealSet. These are instances with a large number of lengthy strings taken from DNA sequences. We have considered 6 DNA datasets, each one comprising 10 different instances. Instances on each dataset are characterized by a number of strings (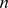) of the same length (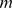). Table shows statistical values (mean (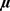) and standard deviation (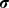)) for relative percentage difference (RPD) from best-known solutions of solutions obtained by each algorithm. n/a indicates that corresponding algorithm was not able to produce a solution to instances in dataset within allowed execution time.

**Table 4:** MPBS and IBS comparison for DNA LargeRealSet instances.

Inst.	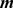	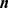	MPBS Impr.	P( 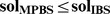 )	IBS Time 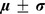	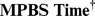 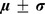
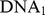	100	100	1.90	100%	0.14±0.00	0.38±0.58
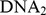	100	500	1.38	100%	0.64±0.01	2.46±3.82
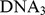	500	100	0.81	100%	0.75±0.01	3.45±5.34
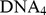	500	500	0.58	100%	3.16±0.01	11.94±15.06
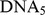	1000	100	0.45	100%	1.65±0.02	12.55±15.36
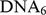	1000	500	0.48	100%	6.80±0.11	22.66±32.03

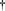
Time(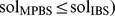.

Fourth column shows mean improvement of solutions obtained by MPBS with respect to the ones obtained by IBS for DNA instances in LargeRealSet. Fifth column shows the percentage of MPBS runs that were able to reach a solution better than or equal to the one provided by IBS. Sixth column shows statistical values (mean (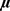) and standard deviation (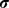)) for the time (in seconds) needed by IBS to produce its solution. Seventh column shows statistical values (mean and standard deviation) for the time (in seconds) needed by MPBS to produce a solution better than or equal to the one provided by IBS.

**Figure 6:**
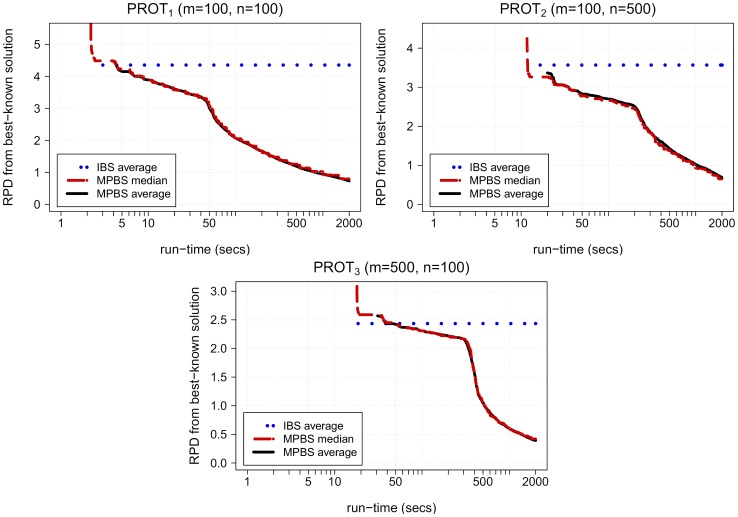
Solution quality over time for different protein datasets in LargeRealSet. Figure shows evolution along execution time of statistical values (average and median) for relative percentage difference (RPD) from best-known solutions of solutions obtained by MPBS algorithm. Average solution obtained by IBS is also shown. Note that the value of solution is considered to be 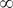 before an algorithm has provided its first solution, hence beginnings of average curves indicate the moment at which at least one solution has been obtained for all executions and instances in the dataset.

For protein instances in LargeRealSet (see Tables 5–6 and Figure 6 ), we allowed a maximum execution time of 2000 seconds per run. Although execution times are larger than the ones allowed for DNA instances, PBS algorithm was not able to produce solutions for these instances. Alphabet sizes are larger and this increases the number of tentative solutions that are produced on each iteration of beam search algorithm. MA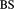 was able to produce solutions for all but the last subset of instances, and remaining algorithms were able to solve all instances. Again, best results are obtained by MPBS followed by IBS. In this case, results produced by MPBS were statistically significant (at the standard 5% level) with respect to the ones produced by IBS in all cases. It can be observed that time to reach IBS solutions increases for these instances, especially for the last subset. Note also that the improvement of solution quality is larger for the first two subsets if we compare them to the ones with same number of string and lengths in DNA instances. If we consider only solutions obtained within 500 seconds of execution, the improvement of MPBS over IBS for 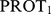 is 2.77% (larger than the one for 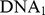 after same execution time), but the one for 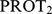 decreases to 1.49%, and this is similar to the one for 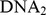.

**Table 5:** Experimental results for protein LargeRealSet instances.

Inst.	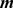	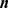	MA 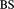 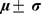	PBS 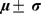	DR 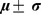	IBS 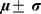	MPBS 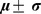
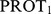	100	100	10.62±1.93	n/a	16.52±2.45	4.36±0.48	0.73±0.38
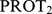	100	500	7.18±2.14	n/a	13.41±3.61	3.57±0.45	0.69±0.46
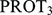	500	100	n/a	n/a	16.10±5.33	2.43±0.27	0.39±0.20

Results obtained by MA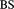, PBS, DR, IBS and MPBS for protein instances in LargeRealSet. These are instances with a large number of lengthy strings taken from protein sequences. We have considered 3 protein datasets, each one comprising 10 different instances. Instances on each dataset are characterized by a number of strings (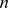) of the same length (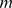). Table shows statistical values (mean (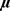) and standard deviation (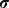)) for relative percentage difference (RPD) from best-known solutions of solutions obtained by each algorithm. n/a indicates that corresponding algorithm was not able to produce a solution to instances in dataset within allowed execution time.

**Table 6:** MPBS and IBS comparison for protein LargeRealSet instances.

Inst.	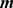	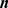	MPBS Impr.	P( 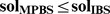 )	IBS Time 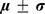	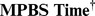 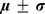
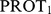	100	100	3.63	100%	1.88±0.07	6.64±7.31
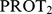	100	500	2.88	100%	10.72±0.42	27.40±42.99
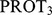	500	100	2.04	100%	12.17±0.30	118.54±134.89

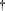
Time(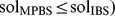.

Fourth column shows mean improvement of solutions obtained by MPBS with respect to the ones obtained by IBS for protein instances in LargeRealSet. Fifth column shows the percentage of MPBS runs that were able to reach a solution better than or equal to the one provided by IBS. Sixth column shows statistical values (mean (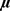) and standard deviation (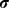)) for the time (in seconds) needed by IBS to produce its solution. Seventh column shows statistical values (mean and standard deviation) for the time (in seconds) needed by MPBS to produce a solution better than or equal to the one provided by IBS.

To sum up, it can be stated that IBS and MPBS algorithms provide best solutions on all instances sets that have been considered. IBS is able to produce high quality solutions within short execution times, but it is not an anytime algorithm and cannot be used to further improve those solutions. MPBS provides best solutions overall but it requires longer execution times than IBS. For very large instances, improvements in solution quality are moderate and execution times increase significantly. This is due to the execution costs of PBS_Perturbation and PBS_Reduction local search procedures that have to solve many different instances of the problem.

### Sensitivity Analysis

With the aim of better understanding the influence of settings for different parameters in the performance of MPBS algorithm, a sensitivity analysis has been performed. For this analysis, we used instances in RandomSet benchmark. All parameters were fixed to the same settings as reported in the previous section (i.e. 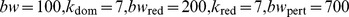 and 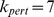), except the one being analysed in each case.

**Figure 7:**
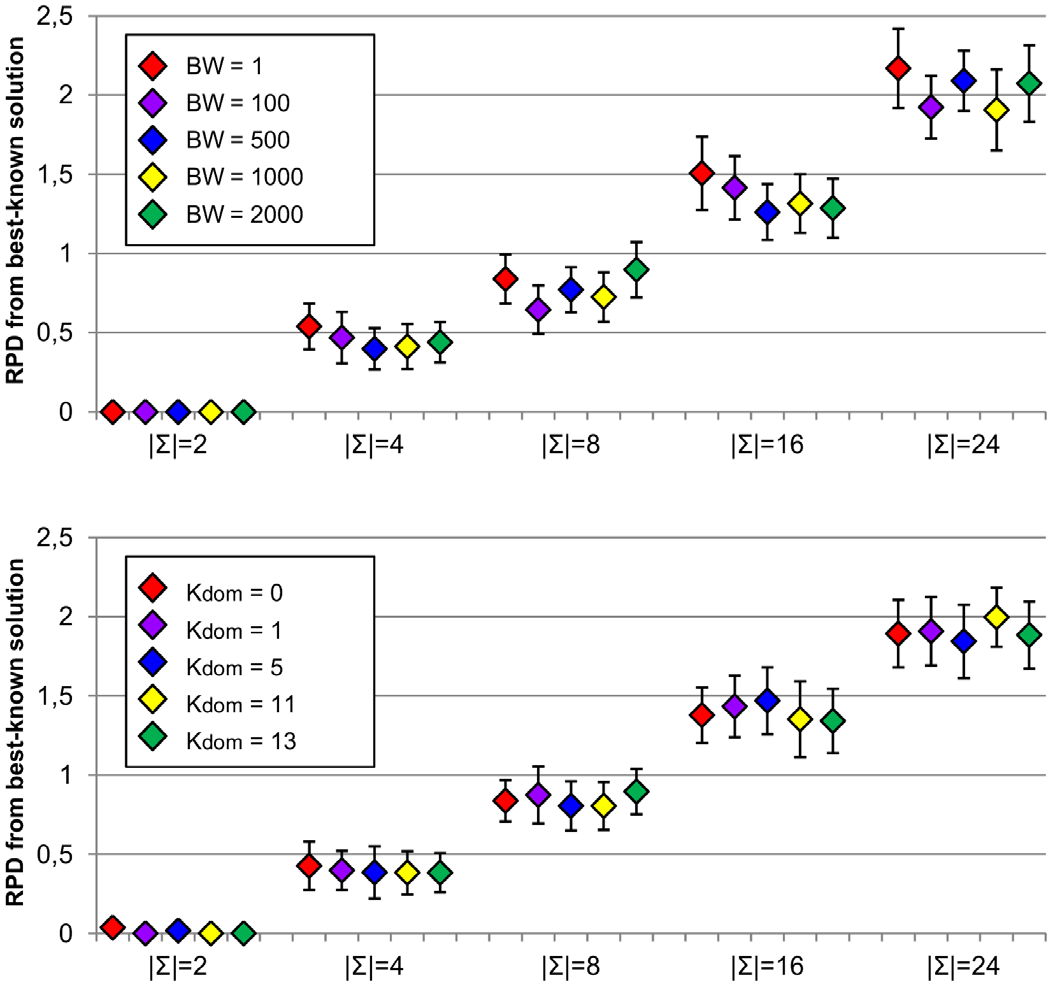
Sensitivity analysis for 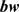 **(top) and** 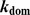 **parameters (bottom).** Figure shows average relative percentage difference (RPD) from best-known solutions along with corresponding confidence intervals at the 95% confidence level obtained by running MPBS algorithm with different settings for 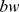 (beam width) and 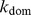 (number of dominators) parameters for the initial construction of solution phase. Experiments were carried out on RandomSet instances with different alphabet sizes (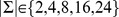).

First of all, we examined the influence of beam width parameter that is used in the construction of initial solutions. For this purpose, 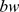 was set to different values (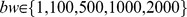). Average RPD from best-known solutions for different alphabet sizes along with corresponding confidence intervals at the 95% confidence level are shown in Figure 7 (top). Also, different settings for the number of dominators used during construction of initial solutions (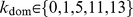) were analysed and results are shown in Figure 7 (bottom). Only small differences in the average quality of solutions are observed. Moreover, confidence intervals overlap considerably and so we cannot conclude from this analysis the existence of significant differences on the average solution qualities obtained by using different settings for these parameters. It should be recalled that these parameters only affect the first phase of the algorithm which produces an initial solution to the problem, and these experiments indicate that all analysed settings are able to provide good starting solutions for the algorithm.

**Figure 8:**
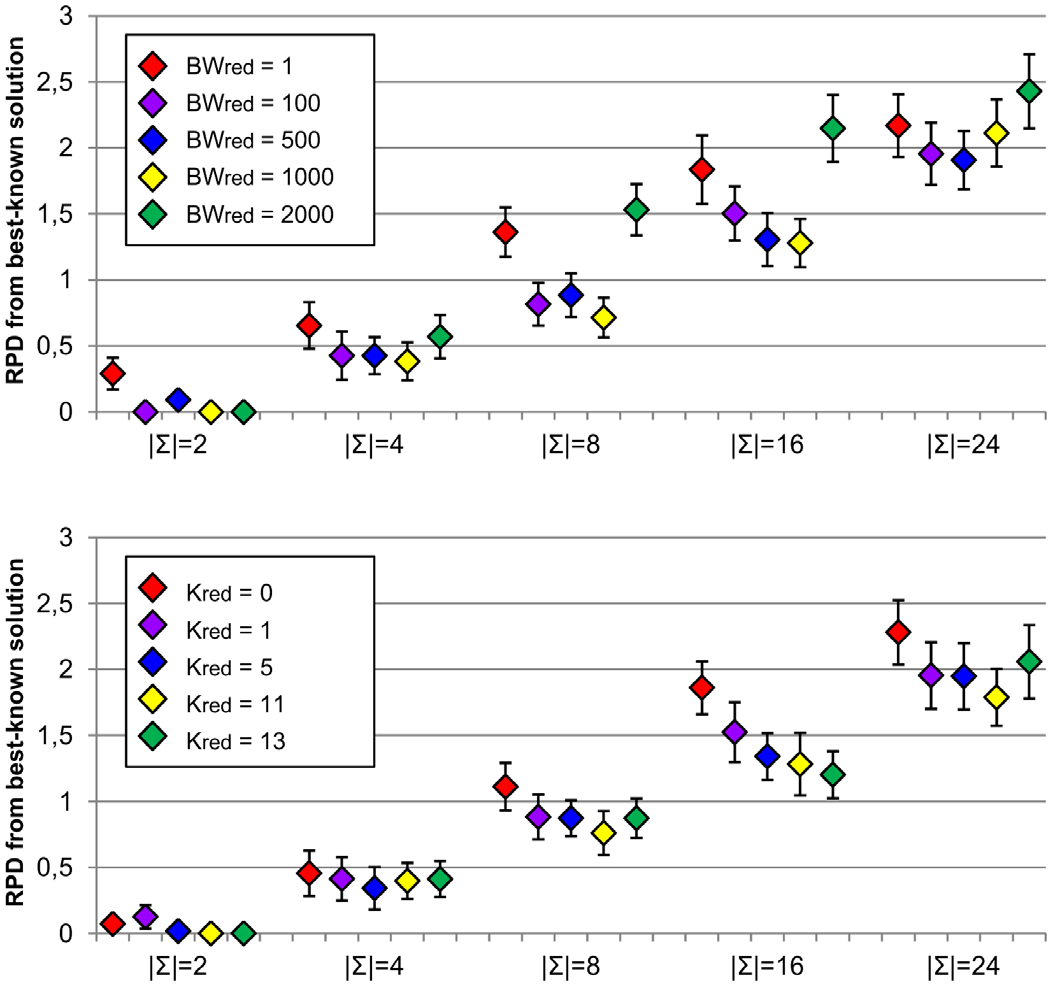
Sensitivity analysis for 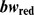 **(top) and** 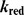 **parameters (bottom).** Figure shows average relative percentage difference (RPD) from best-known solutions along with corresponding confidence intervals at the 95% confidence level obtained by running MPBS algorithm with different settings for 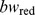 (beam width) and 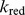 (number of dominators) parameters for the reduction of solution phase. Experiments were carried out on RandomSet instances with different alphabet sizes (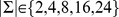).

Results for analysis of 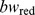 and 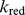 parameters (beam width and number of dominators used in reduction algorithm) are shown in Figure 8. For 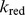 parameter, we can observe that results obtained by the algorithm with 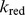 = 11 tend to be better than those obtained without using dominators, especially for larger alphabet sizes. For 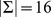, 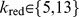 are also better than 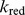 = 0. Results with various number of dominators are not significantly different, except for 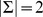, where 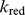 = 1 performs slightly worse than remaining settings. For 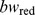 parameter, 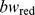 = 1 and 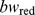 = 2000 tend to produce worse results, whereas for the rest of configurations, no differences can be observed.

**Figure 9:**
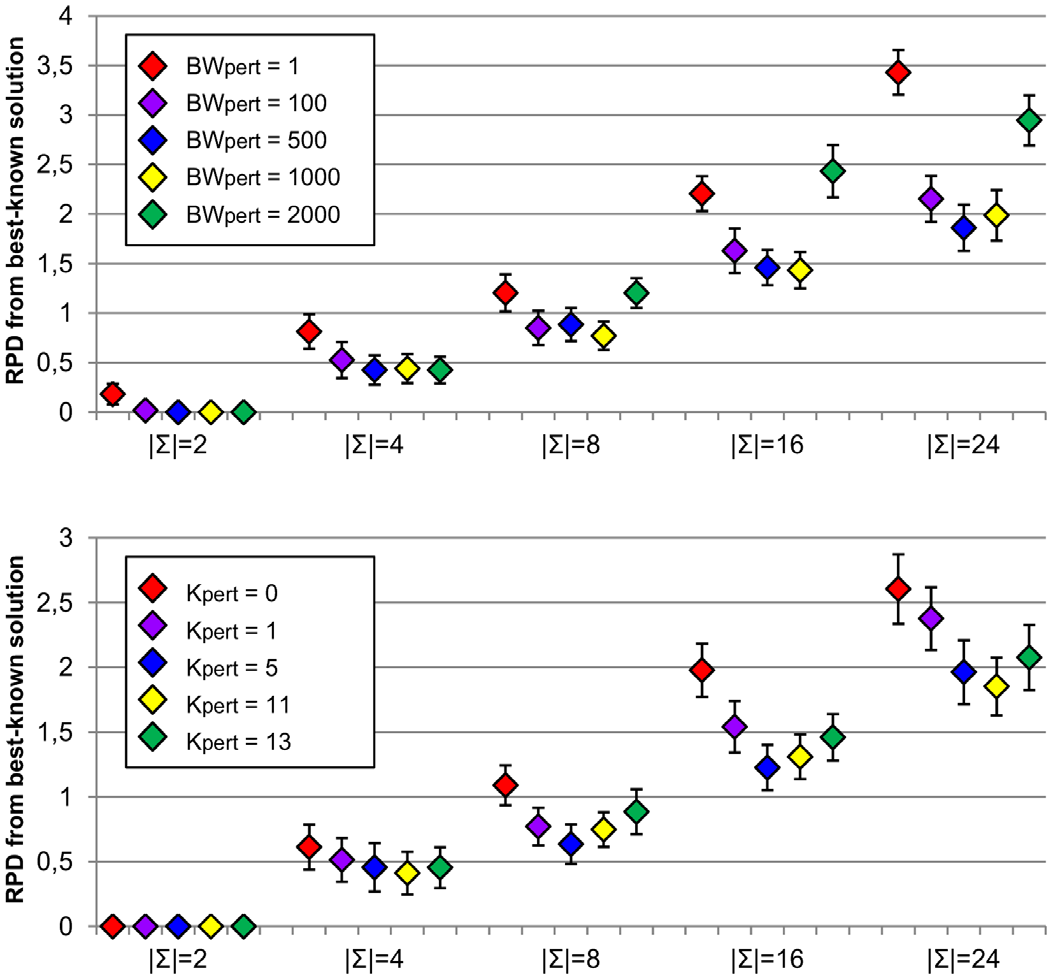
Sensitivity analysis for 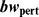 **(top) and** 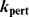 **parameters (bottom).** Figure shows average relative percentage difference (RPD) from best-known solutions along with corresponding confidence intervals at the 95% confidence level obtained by running MPBS algorithm with different settings for 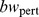 (beam width) and 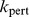 (number of dominators) parameters for the perturbation of solution phase. Experiments were carried out on RandomSet instances with different alphabet sizes (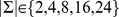).

Figure 9 shows results for different settings of 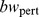 and 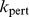 (used in perturbation algorithm). For 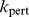 parameter, using dominators is beneficial when 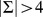 most of the time. There are not significant differences when using different numbers of dominators, except for 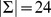, where 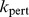 = 1 is worse than 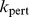 = 11. For 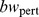 parameter, 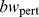 = 1 and 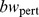 = 2000 produce worse results in most cases, and differences in average solution quality are considerable for largest alphabet sizes.

**Figure 10:**
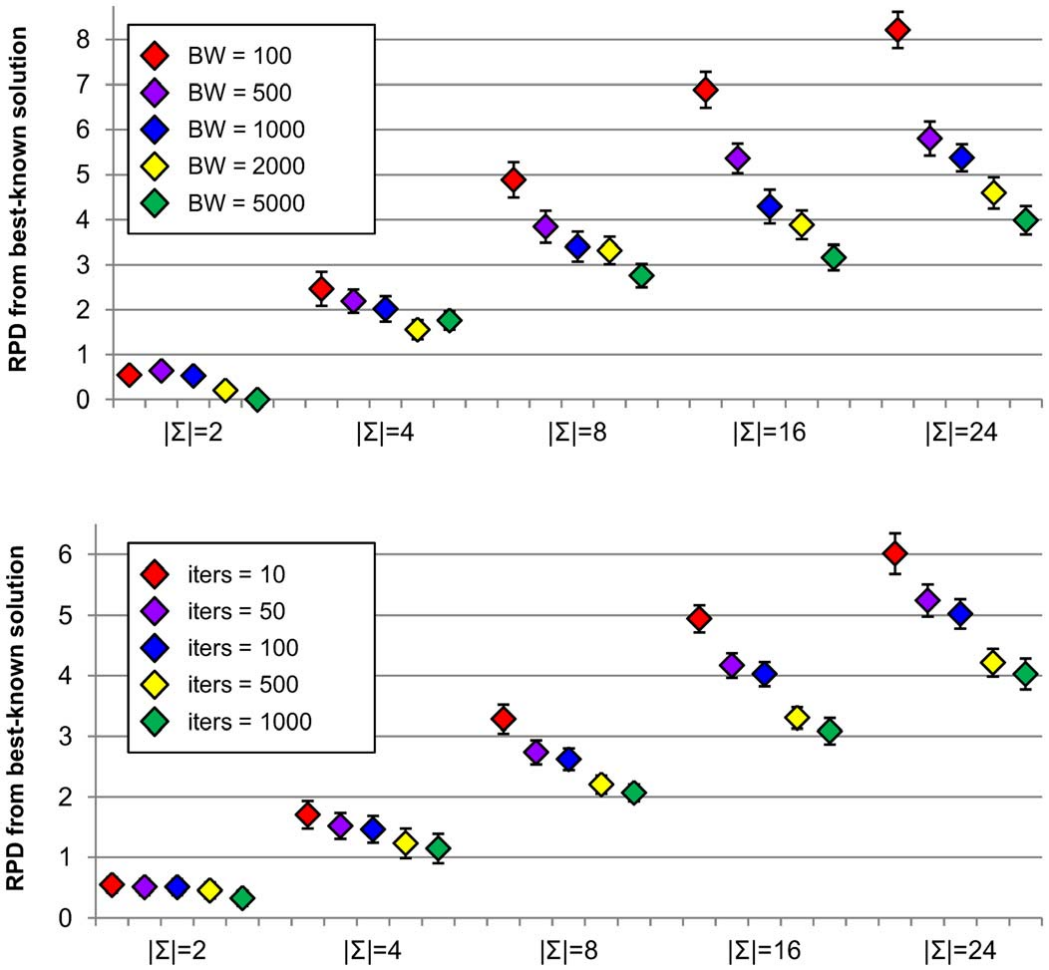
Sensitivity analysis for Probabilistic Beam Search algorithm considering different beam widths (top) and executions with different number of iterations (bottom). Figure shows average relative percentage difference (RPD) from best-known solutions along with corresponding confidence intervals at the 95% confidence level obtained by running a single iteration of PBS algorithm with different beam widths and for best solution found after executing different number of iterations of PBS algorithm (with a fixed beam width of 100). Experiments were carried out on RandomSet instances with different alphabet sizes (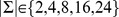).

In conclusion, results obtained by the algorithm are very similar for the range of settings that we analysed for 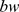 and 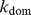 parameters. In addition, for 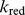 and 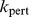 parameters, almost no significant differences can be observed if at least one dominator is used. For 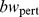 and 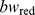 parameters, extreme settings tend to produce worse results than remaining ones, for which results are similar, although settings with different orders of magnitude were considered. With the aim of explaining these results, we analysed the behaviour of constituent Probabilistic Beam Search algorithm for different beam widths. To this end, Figure 10 (top) shows average RPD from best-known solutions for results obtained by running a single iteration of PBS algorithm using different beam widths. It can be observed that the quality of solutions improves consistently by increasing the beam width, especially for instances with larger alphabet sizes. However, execution times also increase with larger beam widths. We also studied the effect of performing different number of iterations of PBS algorithm on the quality of produced solution. Figure 10 (bottom) shows average RPD from best-known solutions of best solution obtained after executing different number of iterations of PBS algorithm with fixed beam width (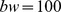). It can be observed that the quality of final solution also increases by doing more iterations. When running MPBS with larger beam widths, solutions obtained by each iteration of PBS algorithm should be better, but less number of iterations can be performed within allowed execution time. On the other hand, decreasing the beam width should produce worse quality solutions on each iteration but a greater number of iterations can be performed, and this also leads to improved solutions. Within a range of different settings for beam width parameter, being able to do more iterations seems to compensate for using smaller beam widths. For 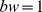, the performance of PBS algorithm degrades, and being able to perform more iterations does not allow MPBS algorithm to achieve good quality solutions. For very large beam widths, execution time needed by PBS algorithm grows excessively and, as a consequence, MPBS performance also gets worse.
